# A blood RNA transcript signature for TB exposure in household contacts

**DOI:** 10.1186/s12879-020-05116-1

**Published:** 2020-06-09

**Authors:** Philip Kam Weng Kwan, Balamurugan Periaswamy, Paola Florez De Sessions, Wenwei Lin, James S. Molton, Claire M. Naftalin, Ahmad Nazri Mohamed Naim, Martin L. Hibberd, Nicholas I. Paton

**Affiliations:** 1 Department of Medicine, Yong Loo Lin School of Medicine, National University of Singapore, NUHS Tower Block Level 10, 1E Kent Ridge Road, Singapore, 119228 Singapore; 2 Genome Institute of Singapore, Agency for Science, Technology and Research, Singapore, Singapore; 3 London School of Hygiene & Tropical Medicine, London, UK; 4 Department of Microbiology and Immunology, Yong Loo Lin School of Medicine, National University of Singapore, Singapore, Singapore

**Keywords:** Tuberculosis; gene expression, Biomarkers, TB exposure, TB infection; RNA sequencing, Whole blood

## Abstract

**Background:**

Current tools for diagnosing latent TB infection (LTBI) detect immunological memory of past exposure but are unable to determine whether exposure is recent. We sought to identify a whole-blood transcriptome signature of recent TB exposure.

**Methods:**

We studied household contacts of TB patients; healthy volunteers without recent history of TB exposure; and patients with active TB. We performed whole-blood RNA sequencing (in all), an interferon gamma release assay (IGRA; in contacts and healthy controls) and PET/MRI lung scans (in contacts only). We evaluated differentially-expressed genes in household contacts (log2 fold change ≥1 versus healthy controls; false-discovery rate < 0.05); compared these to differentially-expressed genes seen in the active TB group; and assessed the association of a composite gene expression score to independent exposure/treatment/immunological variables.

**Results:**

There were 186 differentially-expressed genes in household contacts (*n* = 26, age 22–66, 46% male) compared with healthy controls (*n* = 5, age 29–38, 100% male). Of these genes, 141 (76%) were also differentially expressed in active TB (*n* = 14, age 27–69, 71% male). The exposure signature included genes from inflammatory response, type I interferon signalling and neutrophil-mediated immunity pathways; and genes such as BATF2 and SCARF1 known to be associated with incipient TB. The composite gene-expression score was higher in IGRA-positive contacts (*P* = 0.04) but not related to time from exposure, isoniazid prophylaxis, or abnormalities on PET/MRI (all *P* > 0.19).

**Conclusions:**

Transcriptomics can detect TB exposure and, with further development, may be an approach of value for epidemiological research and targeting public health interventions.

## Background

Following exposure to and infection with tuberculosis (TB) many individuals enter a state of latent TB infection (LTBI) [1, 2]. An estimated 25% of the world’s population has LTBI, with the highest prevalence found in the WHO Southeast Asia (31%) and Western Pacific regions (28% overall, 13% in Singapore), compared to 11–22% in the other regions of the world [3–5]. LTBI forms a large reservoir from which new active TB cases develop. Prevention of transmission and consequent replenishment of the LTBI reservoir and treatment of LTBI to prevent development of active disease are essential if the goals of the WHO’s End TB are to be achieved [6].

Current tools for diagnosis of LTBI that depend on detection of an immune response to exogenously-administered TB antigens - in vivo as the tuberculin skin test (TST), or ex vivo as an interferon gamma release assay (IGRA) [7] - detect immunological memory of past exposure but are unable to determine whether exposure is recent. A test that could identify recent exposure may be of value for epidemiological research as well as for public health, identifying areas with ongoing high rates of transmission where active case-finding and infection control measures could be enhanced to prevent new cases of LTBI. More than half of prevalent culture positive TB disease is asymptomatic, and much transmission goes unrecognised [8, 9].

Transcriptome-based signatures have been shown to detect incipient disease in individuals with LTBI that precedes the onset of clinical disease, indicating the potential of transcriptomics to respond dynamically to subclinical events in the TB disease spectrum [10–12]. PET-based imaging studies have demonstrated the existence of early changes, mainly in lymph nodes, following exposure to TB, [13, 14] and this suggests that there may be sufficient disease activity early after exposure to drive a transcriptome response. In this study, we aimed to determine whether a transcriptome signature can be identified that differentiates individuals with recent TB exposure from those without; to explore the biological basis of the early exposure signature; and to examine the relationship between the signature and relevant clinical and demographic factors.

## Methods

### Participants and sample collection

We performed a cross-sectional study, comparing gene expression patterns in three participant groups: a group with recent TB exposure, without active clinical TB (household contact group); group without recent TB exposure or active clinical TB (healthy control group, as a reference to determine differential gene expression in the TB exposed); and a group of patients with clinical TB (active TB group; to provide context for the TB exposure signature).

The household contact group comprised 26 adult contacts of smear-positive index TB cases. They had participated in a PET-based imaging study conducted at the National University Hospital (NUH), Singapore from 2013 to 2015, for which the main eligibility criteria were age over 21 years; residing in the same house as a newly-diagnosed smear-positive pulmonary TB patient for at least 1 month prior to start of anti-TB treatment in the index case [14–16]; receiving no previous treatment for active TB; having no evidence of active clinical TB; and no severe uncontrolled diabetes (Supplementary Table 1). Demographic, clinical and exposure data were collected. Time from exposure was estimated as the difference between the date that the index patient first reported symptoms or began to live in the same household as the contact, whichever was later, and the date that the blood was drawn for transcriptome profiling. An IGRA test was performed (QuantiFERON-TB Gold In-Tube Test, Qiagen, Hilden, Germany) and repeated after approximately 3 months if the initial test was negative or indeterminate. A whole blood sample was drawn into a Tempus RNA preservation tube (Thermo Fisher Scientific, Massachusetts, USA), frozen within 2 h of collection, and stored at − 80 °C until analysis. A PET/MRI scan was performed, as previously described [14].

The healthy control group comprised 5 volunteers (recruited from a database) who had previously participated in a PK/PD study at NUH from 2015 to 2016. Participants with immune deficiency, past history of TB, or known contact with a TB index case in the previous year were not enrolled (Supplementary Table 1). Participants were assessed clinically (symptom questionnaire) to rule out active TB; demographic and clinical data were collected; an IGRA test was performed; and whole blood collected in an RNA preservation tube (as above).

The active TB group comprised 14 patients who had a clinical diagnosis of pulmonary TB (presence of symptoms, chest X-ray findings and positive GeneXpert; with later microbiological confirmation); and less than 1 week of standard combination treatment (Supplementary Table 1). They had participated in a PET-based imaging study at NUH from 2013 to 2015. No IGRA testing was performed. A whole blood sample was collected in an RNA preservation tube and stored (as above).

All study protocols were approved by the Singapore National Healthcare Group Domain Specific Review Board (NHG DSRB) and all participants gave written informed consent.

### Extraction of total RNA from peripheral blood and RNA sequencing

Samples were thawed, total RNA extracted, and DNAse-treatment was performed using the column-based Tempus™ Spin RNA Isolation kit (ThermoFisher Scientific, Massachusetts, USA). RNA was quantified using the Agilent 2100 Bioanalyzer (Agilent Technologies, California, USA). cDNA libraries were constructed using the TruSeq total RNA V2 (Illumina, USA). Total RNA was reverse transcribed into cDNA and ligated with RNA sequencing adaptors. RNA sequencing was performed on Illumina HiSeq Rapid v2 (2x76bp) at the Genome Institute of Singapore (GIS).

### RNASeq data analyses and functional annotation

Sequenced reads (paired-end FASTQ files) were mapped to the Genome Reference Consortium Human Build 38 release 86 (GRCh38.r86) by using STAR aligner [17]. The aligned reads were counted for each gene using HTSeq [18]. Sample read counts were adjusted for library size and normalized using Trimmed Mean of M-values (TMM) method and multidimensional scaling plots were used to detect any outlier samples (none found) using Bioconductor package EdgeR [19].

Differential gene expression was assessed using the exact test in EdgeR. Genes were considered differentially expressed if they had a false-discovery rate (FDR, Benjamini-Hochberg) [20] of less than 0.05 and at least a log2 fold change of ±1.

Gene expression in the household contact group was compared with the healthy control group to identify differentially expressed genes (the “TB exposure signature”). The nature of the TB exposure signature was explored further by creating a comprehensive protein network representation of the proteins associated with the genes in the signature using Search Tool for the Retrieval of Interacting Genes/Proteins (STRING) [21]. Analysis of the associated functional pathways was performed using the GeneOntology (GO) Biological Processes on STRING.

The probability of identifying overlap genes in the TB exposure signature with genes overexpressed in active TB (compared with the same control group; using the same FDR thresholds) was tested by exact hypergeometric probability method [22]. The association between the relative magnitudes of overexpression of genes in the two groups was assessed by Pearson’s correlation and the difference by Wilcoxon signed-rank test.

An exposure risk score to quantify the strength of the TB exposure signature was calculated for each individual in the household contact group using the normalized gene expression values of the genes in the TB exposure signature, following an approach described previously [23–25]. Within the household contact group, the relationship between the exposure risk score and exposure variables - time from first exposure (at least 60 days versus less than 60 days), use of isoniazid prophylaxis at the time of the blood draw, IGRA positivity, and presence of abnormalities on PET/MRI scan - was assessed by Mann-Whitney U test.

The area under the curve (AUC) of the receiver operating characteristic (ROC) curve obtained with the exposure risk score from the TB-exposed household contacts and TB unexposed healthy control groups was calculated using a parametric method and the optimal cut-off for distinguishing between those with and without exposure was determined (ROCR package) [26].

All analyses and figures were generated using the R software or custom Python scripts.

The sample size for household contacts was determined by pragmatic considerations of the availability of whole-blood RNA-preserved samples from previous household contact studies using PET-MRI performed in our group, assay costs and the exploratory nature of the study that did not intend to validate the signature against disease-progression outcomes; the sample size for the healthy control group and active TB group were based on similar pragmatic considerations and experience with other studies where comparison groups of this size typically allow detection of differential gene expression in a study group of interest.

## Results

The 26 participants in the *household contact group* (46% male, mean age 42 years, 46% Chinese ethnicity) lived in 16 discrete households (ten households contributed one contact; three households, two contacts; two households, three contacts; one household, four contacts). They were studied at a median of 95 (range 0–752) days from estimated first exposure to infection. Thirteen (50%) were IGRA positive and 4 (15%) had started isoniazid treatment a median of 17 days (range 7–44 days) prior to the study day; 7 had abnormalities (mainly lymphadenopathy) observed on PET/MRI scan, as previously reported [14]. Participants were otherwise well, without any significant underlying medical conditions.

Of the 5 participants in the *control group* (100% male, mean age 29 years, 100% Chinese ethnicity), two were IGRA positive (no recent exposure history). Participants did not have any significant underlying medical conditions.

The 14 participants in the *active TB* group (71% male, mean age 51 years, 40% Chinese ethnicity) all had drug-susceptible TB and had been on treatment for a median of 5 days (range 2–21 days) on the day of the study. Seven (50%) were smear positive; 7 (50%) had cavitation on the chest-X-ray; 5 (36%) still had fever and 8 (57%) cough at the time of the study. Six of the patients had diabetes mellitus.

We found 186 genes that were differentially expressed (180 induced, 6 repressed) in household contacts compared to healthy controls (listed in Supplementary Table 2). Of these 186 genes, 141 (76%) were also differentially expressed in the patients with active TB (versus healthy controls); there was a moderate association between the relative magnitude of expression of individual genes in the two groups (r = 0.66, *P* < 0.0001) although overall expression values were lower in the household contacts (*P* < 0.00001; Supplementary Figure 1).

Of these 186 genes, 69 have been reported previously in published human TB-related signatures (Supplementary Table 3), including 68 previously reported for active TB (including CD274, IRF7, IFI6) [23–25, 27–35]; 5 in a 126-gene signature of LTBI (ADM, DUSP2, IER3, OSM, SOCS3; *P* < 0.011 for overlap) [33]; 4 in a 16-gene signature of incipient TB in those with LTBI (ANKRD22, BATF2, SERPING1, SCARF1; *P* < 0.001 for overlap), [10] and 2 in a 3-gene signature of incipient TB in TB contacts (BATF2 and SCARF1; *P* < 0.0001) [36].

**Fig. 1:**
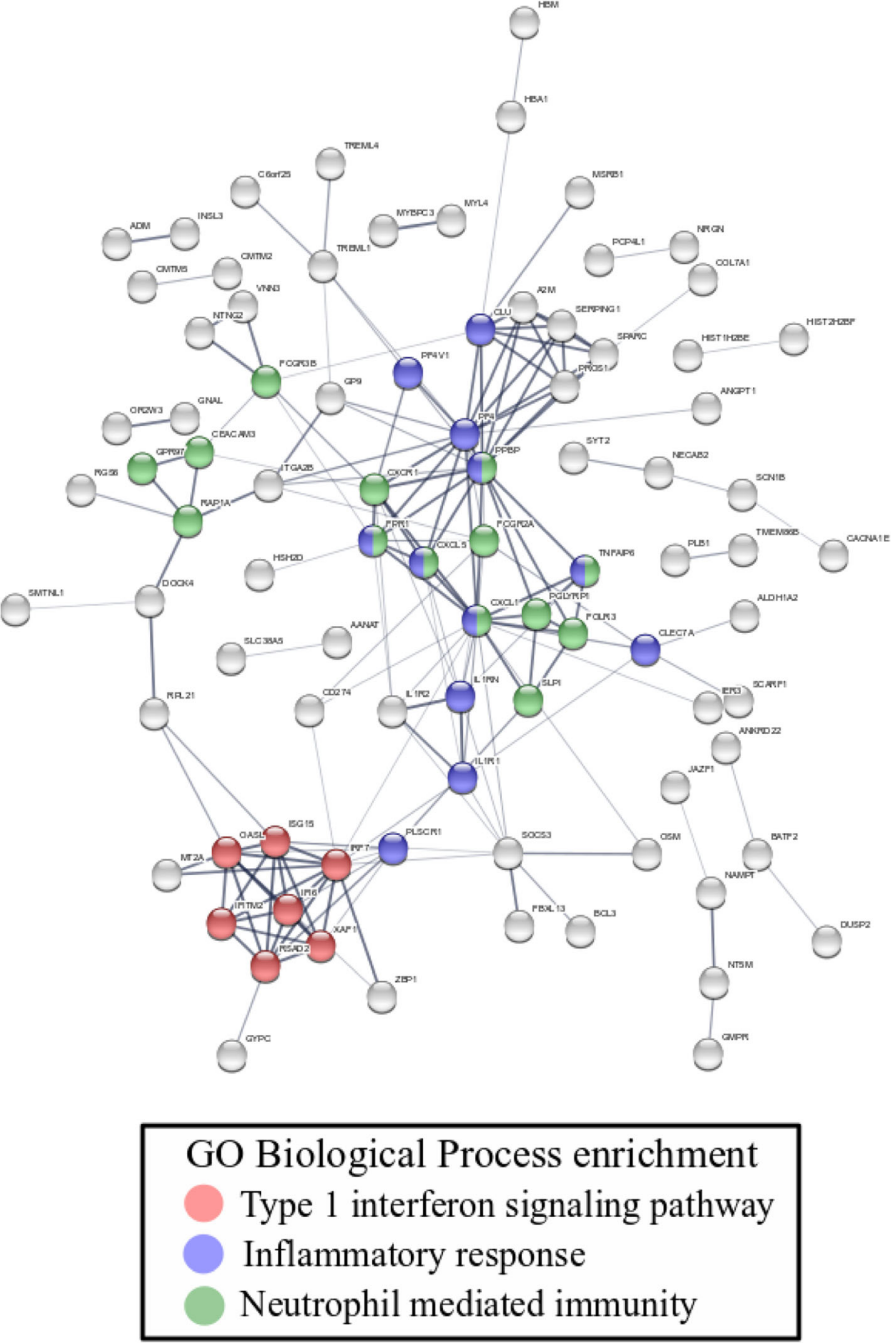
Protein-protein network of the 186 exposure genes. Each circle represents a protein encoded by a gene. Colors were assigned proteins that belong to three overrepresented pathways relevant to TB infection. Type 1 interferon signaling pathway (red circles: IFI6, IFITM2, IRF7, ISG15, OASL, RSAD2, XAF1); Inflammatory response (blue circles: CLEC7A, CLU, CXCL1, CXCL5, FFAR2, FPR1, IL1R1, IL1RN, PF4, PF4V1, PLSCR1, PPBP, TNFAIP6, TPST10); Neutrophil mediated immunity (green circles: ANXA3, CEACAM3, CXCL1, CXCL5, CXCR1, FCGR2A, FCGR3B, FOLR3, FPR1, GPR97, PGLYRP1, PPBP, RAP1A, SLPI, TNFAIP6)

Analysis of the proteins associated with all 186 genes and functional analysis of protein pathways revealed clusters of proteins from multiple immune response pathways including the inflammatory response, type I interferon signalling and neutrophil-mediated immunity pathways (Fig. 1, Supplementary Table 4).

The TB exposure risk score, a composite score calculated from the values of expression of the 186 genes, did not differ by time from first exposure (median 17,758 [11,543-31,650] versus 27,005 [10,618-62,190] in those with exposure onset ≤60 days versus > 60 days prior to sampling respectively, *P* = 0.29); by use of isoniazid prophylaxis (median 38,432 [16,518 – 62,190] versus 22,758 [10,618 to 44,448] in those taking versus not taking isoniazid prophylaxis respectively; *P* = 0.197); or by presence of lung abnormalities on PET/MRI scan (median 21,400 [15,836 to 43,919] versus 26,510 [10,618 to 62,190] in those with PET/MRI abnormalities versus no abnormalities respectively; *P* = 0.955).

However, the risk score was higher in contacts who were IGRA positive (median 28,028 [15,836 – 62,190]) versus IGRA negative (17,469 (10,618 – 44,448]; *P* = 0.044). The risk score was positive (above the threshold identified on ROC analysis) in 23 of 26 (88%) contacts, of whom only 13 (56%) were IGRA positive.

## Discussion

We identified a 186-gene signature that can differentiate people with recent exposure to TB from those without recent exposure. Transcriptomic signatures have been shown previously to differentiate active TB from LTBI, [23, 25, 28, 29, 32, 33, 35, 37] from other infections, [23, 24, 27, 38] and from healthy individuals [25, 27, 28, 30, 32, 33]; to differentiate LTBI from those who are uninfected [33, 34, 39]; to identify those at risk of relapse after treatment for TB disease [40]; and to identify those with LTBI (including household contacts) who are at risk of reactivation [10, 11, 25, 36]. However, this is the first time transcriptomics has been used to differentiate those with and without recent TB exposure. Our study adds to the potential applications for transcriptomics-based testing in an area of special need given the limitations of current diagnostic tools in early TB disease.

This signature is biologically plausible as a signature of TB exposure. The majority of genes were also found in our patients with active TB as well as many published active-TB signatures. Several genes are highly specific for TB (i.e. CD274, IRF7, IFI6) [27] and others have been found in signatures indicative of incipient/subclinical TB (such as BATF2, SCARF1 and SERPING1) [10, 36]. Many of the genes in the signature are non-specific inflammatory markers and overexpression might reflect recent exposure to respiratory or other circulating infections such as dengue fever that are common in Singapore. However, this is unlikely to explain our findings because of the strong epidemiological evidence for recent exposure to a case of smear-positive TB and the low probability that the majority of household contacts were exposed to viral infections within a short period prior to blood sampling. Furthermore, analysis of functional protein pathways revealed patterns characteristic of TB (type I interferon signalling, neutrophil mediated and inflammation responses) [27] and was consistent with the evolution of transcriptome signatures in macaques followed from time of infection (up-regulation of inflammation and interferon pathways was seen during the early phases post infection) [8]. Although interferon pathways are also evident in viral infections, the overall pattern of pathway activation is distinct from that seen in patients with common respiratory viral infections such as respiratory syncytial virus and influenza virus, in which Notch signalling [41] and ubiquitination signaling pathways [42] respectively, are distinctive host responses.

Although we did not find a relationship between the exposure signature and time from exposure, isoniazid prophylaxis, or the presence of lung PET/MRI abnormalities, this may simply reflect the small sample size and imprecision of estimates. The transcriptome measurements were made at a single timepoint, whereas studies in macaques indicate a dynamic situation with progressive attenuation after about 2 months following exposure [8]. Time from exposure was derived from subjective estimate of the onset of symptoms by the index case, and exposure likely comprises a series of repeated exposures over a prolonged period within a household prior to diagnosis and treatment of the index case. Isoniazid effects on bacterial replication may not directly affect the magnitude of the host immune response if this evolves independently of the underlying bacterial insult [43]. The time course of development and resolution of structural/metabolic changes in the PET/MRI is also unknown and may not follow the same pattern as those of the immune or inflammatory processes.

The finding of a higher TB exposure risk score in IGRA positive contacts provides additional support for validity of our signature. The positive transcriptome score in ten participants who were IGRA negative may reflect clearance of infection by innate immune responses preventing sufficient TB antigen exposure to stimulate memory T-cell responses (detected by IGRA). Alternatively, the T-cell responses could be present but below the threshold of detection of IGRA; up to 20% of people with active TB have a negative IGRA, [44] and the test has substantial variability, even for LTBI [45, 46]. It appears that our transcriptome signature is more sensitive than IGRA for detecting exposure, consistent with the finding of expression of a 20-gene signature of active TB in 26% of persistently IGRA-negative TB contacts (followed for up to 12 months after exposure) [25].

A test for recent TB exposure may be of value for TB control programmes or epidemiological researchers seeking to monitor ongoing TB exposure in the community. Traditional tests that require the administration of exogenous antigen at the time of the test (intra-dermal in the case of the TST or in vitro in the case of IGRA) measure durable immune responses that are largely independent of the timing of the original natural exposure [47]. Identifying recent natural (repeat) exposure is especially difficult in a high-burden setting where the background prevalence of IGRA or TST positivity is high. In contrast, the in vivo immune response to naturally-occurring infection measured by a transcriptome test should abate when infection is cleared and may therefore provide more precise temporal information of recent exposure, including repeat exposure in a high-burden setting. Such a test may be of value to identify environments and populations where there are high rates (‘hot spots’) of TB exposure and transmission and where active case-finding and infection control measures could be enhanced: more than half of prevalent culture positive TB disease is asymptomatic, and much transmission goes unrecognised [8, 9].

A transcriptome signature also has the potential to improve the selection of patients for preventive treatment. Traditional tests have a low positive predictive value for disease progression: IGRA/TST results did not add significantly to a clinical/demographic risk score, [48] and the excess risk associated with a positive versus negative IGRA/TST is relatively modest [49]. Our study was not designed to identify a signature that would predict progression of active TB disease; however our signature contained many of the genes shown to predict disease progression in adolescents in South-Africa and contacts in the UK (two out of the three reported genes, BATF2 and SCARF1) [10, 36]. Our study lends further support to the potential of this approach for application in an Asian population.

Limitations of our study include the relatively small sample size, although it was comparable to other exploratory studies in the field and proved sufficient to identify a signature and analyse associated pathways. We did not collect information on socioeconomic status of contacts and controls that might partially confound the findings, although differences would be unlikely to account for the gene signature in the contacts, given that it included well known TB-associated genes and protein pathways. Our signature, although biologically plausible and consistent with published literature, would require validation in a large independent cohort prior to widespread use for the detection of recent TB exposure. We performed one transcriptome measurement per participant and did not examine evolution of the individual components of the signature over time. A macaque study (where the precise timing of exposure is known) found the greatest differential expression of genes from pre-infection values occurred between 20 to 56 days post-infection, and by day 120 following infection the whole blood signature had returned to baseline values [8]. The median time from estimated exposure to sampling in our study was 95 days which may have missed the peak response in some patients. However, a comprehensive and systematic description of longitudinal changes would require more precise knowledge of the timing of first exposure as well as collection of baseline samples soon after exposure, both of which are challenging.

## Conclusions

We have expanded the scope of application of transcriptomics to identify a signature of recent TB exposure, independent of IGRA testing. Further validation studies and work to optimise the signature are needed, followed by validation of a reduced set of genes by RT-PCR, but this research illustrates the potential for this approach to be applied for screening to identify areas of high TB exposure that could benefit from enhanced case finding and infection control measures; and supports the potential of transcriptomics to identify more precisely the individuals who would benefit from preventive treatment.

## Supplementary information

**Additional file 1 Supplementary Figure 1.** Panel A: Comparison of the 186 differentially expressed genes in contacts with 792 genes differentially expressed genes in patients with active TB (both compared to healthy controls) yielded 141 overlap genes. Panel B: Log_2_fold change expression values of the 141 overlap genes for Contacts and active TB (Wilcoxon signed rank test *p* < 0.00001). **Supplementary Table 1.** Inclusion and exclusion criteria of household contact, active TB and healthy control groups. **Supplementary Table 2.** 186 genes from the comparison of exposed household contacts and healthy control (false discovery rate < 0.05; log2fold change > 1 or < − 1). **Supplementary Table 3.** Genes in the exposure signature in this study that have been reported in other studies with active TB, latent and incipient TB. Numbers in cells are log2 fold change relative to comparison group (negative value represents downregulation); +, gene present but fold change not reported; −, gene not reported in study; *, IGRA positive vs IGRA-negative non-exposed controls; **, IGRA/TST positive progressors vs non-progressors. **Supplementary Table 4.** All overrepresented GO Biological Processes of the protein-protein network from STRING analysis. The three highlighted pathways are represented as colored circles in Fig. 1.

## Data Availability

The datasets generated and/or analysed during the current study are available in NCBI Sequencing Read Archive under the BioProject accession PRJNA595691 (http://www.ncbi.nlm.nih.gov/595691).

## Abbreviations

TB: Tuberculosis
LTBI: Latent TB infection
TST: Tuberculin skin test
IGRA: Interferon gamma release assay
PET/MRI: Positron-emission tomography/Magnetic Resonance Imaging
PK/PD: Pharmacokinetics/Pharmacodynamics
