# Supplementary material for: A blood RNA transcript signature for TB exposure in household contacts
Source: BMC Infect Dis. 2020 Jun 9;20:403. doi: 10.1186/s12879-020-05116-1 (PMC7282166; doi:10.1186/s12879-020-05116-1)
Supplement: Supplementary file 1 — Additional file 1 Supplementary Figure 1. Panel A: Comparison of the 186 differentially expressed genes in contacts with 792 genes differentially expressed genes in patients with active TB (both compared to healthy controls) yielded 141 overlap genes. Panel B: Log2fold change expression values of the 141 overlap genes for Contacts and active TB (Wilcoxon signed rank test p < 0.00001). Supplementary Table 1. Inclusion and exclusion criteria of household contact, active TB and healthy control groups. Supplementary Table 2. 186 genes from the comparison of exposed household contacts and healthy control (false discovery rate < 0.05; log2fold change > 1 or < − 1). Supplementary Table 3. Genes in the exposure signature in this study that have been reported in other studies with active TB, latent and incipient TB. Numbers in cells are log2 fold change relative to comparison group (negative value represents downregulation); +, gene present but fold change not reported; −, gene not reported in study; *, IGRA positive vs IGRA-negative non-exposed controls; **, IGRA/TST positive progressors vs non-progressors. Supplementary Table 4. All overrepresented GO Biological Processes of the protein-protein network from STRING analysis. The three highlighted pathways are represented as colored circles in Fig. 1. [file 12879_2020_5116_MOESM1_ESM.docx]

A


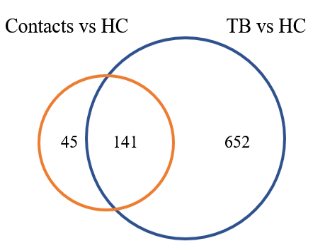


B


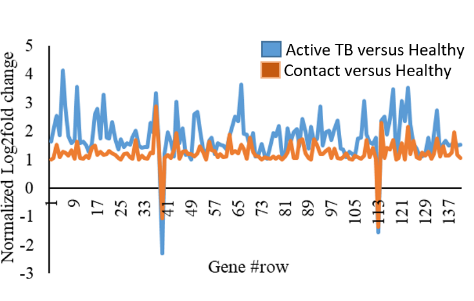


Supplementary Figure 1. Panel A: Comparison of the 186 differentially expressed genes in contacts with 792 genes differentially expressed genes in patients with active TB (both compared to healthy controls) yielded 141 overlap genes. Panel B: Log_2_fold change expression values of the 141 overlap genes for Contacts and active TB (Wilcoxon signed rank test p<0.00001).

Supplementary Table 1. Inclusion and exclusion criteria of household contact, active TB and healthy control groups.

| Group | Inclusion criteria | Exclusion criteria |
| --- | --- | --- |
| Household contacts | 1. Age 21 years or over 2. Close household contact of any patient with smear positive pulmonary TB (close contact defined as sleeping in the same house for at least one month   with a TB patient prior to start of treatment).   1. Willing to comply with the study visits and procedures 2. Willing and able to provide written informed consent | 1. Treated for TB within the past 12 months 2. Cardiac pacemaker, aneurysm clip or other metallic implant considered unsafe for MRI 3. Diabetes that is, in the judgment of the investigator, so poorly controlled that it would prevent adequate PET scanning. 4. Women who are currently pregnant or breastfeeding. |
| Active TB patients | 1. Aged 40 - 70 years of age 2. Clinical diagnosis of pulmonary TB with characteristic symptoms and compatible X-ray findings plus microbiological confirmation with one or more of (i) AFB smear-positive or (ii) molecular test positive or (iii) TB culture positive. Tests done in previous 6 weeks are acceptable for diagnostic purposes. 3. Willing to comply with the study visits and procedures 4. Willing and able to provide written informed consent | 1. Patient should not have taken more than 5 doses of TB medication at the time of the baseline visit) 2. Malignancy requiring chemotherapy or radiotherapy 3. Cardiac pacemaker, aneurysm clip or other metallic implant considered unsafe for MRI 4. Estimated creatinine clearance < 80 ml/min using Cockcroft-Gault formula, based on serum creatinine done any time in the previous 1 week:   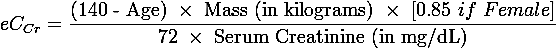  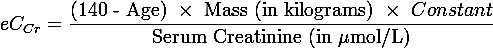   1. Where Constant is 1.23 for men and 1.04 for women. 2. Diabetes that is, in the judgment of the investigator, so poorly controlled that it would prevent adequate PET scanning. 3. Women who are currently pregnant or breastfeeding 4. Occupation involving substantial exposure to radiation 5. History of medical procedures in the last year involving substantial exposure to radiation (>2mSv). |
| Healthy controls | 1. Aged over 21 years and below 60 years old 2. Male or female willing to comply with the study visits and procedures 3. Willing and able to provide written informed consent | 1. Women who are currently pregnant or breastfeeding 2. Body weight below 50kg 3. Clinical signs of active TB in the opinion of the investigator 4. Previous hypersensitivity, intolerance or allergy to rifampicin, pyrazinamide, COX-2 inhibitors, non-steroidal anti-inflammatory drugs or sulfonamides 5. Current use of any drugs or medication known to have an interaction with rifampicin, pyrazinamide or celecoxib 6. Current use or use in the last 2 weeks of non-steroidal anti-inflammatory drugs or COX-2 inhibitors 7. Current use or use in the last 2 weeks of any drugs, over the counter or herbal preparations that are known or potential inhibitors or inducers of cytochrome P450 enzymes 8. Evidence of renal or hepatic dysfunction or any clinically significant deviation from normal during screening including laboratory determinations 9. Known hepatic disease, recent hepatitis (within last 6 months) or alcohol abuse 10. Known cardiovascular disease, heart failure, stroke or current cardiovascular risk factors 11. Known peptic ulcer disease, previous gastrointestinal bleed or current risk factors for gastrointestinal events 12. Acute or previous gout, acute porphyria 13. Any other significant condition that would, in the opinion of the investigator, compromise the volunteer’s safety or outcome in the trial 14. Current participation in other clinical intervention trial or research protocol (participation in other studies that do not involve an intervention may be allowed but this must be discussed and approved by the Principal Investigator) |

Supplementary Table 2. 186 genes from the comparison of exposed household contacts and healthy control (false discovery rate <0.05; log2fold change >1 or <-1).

| Ensembl ID | Gene name | Gene description | Log_2_FC |
| --- | --- | --- | --- |
| ENSG00000005961 | ITGA2B | integrin subunit alpha 2b | 1.23 |
| ENSG00000008438 | PGLYRP1 | peptidoglycan recognition protein 1 | 1.21 |
| ENSG00000017483 | SLC38A5 | solute carrier family 38 member 5 | 1.15 |
| ENSG00000069399 | BCL3 | BCL3 transcription coactivator | 1.07 |
| ENSG00000073150 | PANX2 | pannexin 2 | 1.06 |
| ENSG00000074660 | SCARF1 | scavenger receptor class F member 1 | 1.06 |
| ENSG00000088726 | TMEM40 | transmembrane protein 40 | 1.04 |
| ENSG00000090013 | BLVRB | biliverdin reductase B | 1.16 |
| ENSG00000093134 | VNN3 | vanin 3 | 1.25 |
| ENSG00000099985 | OSM | oncostatin M | 1.23 |
| ENSG00000101460 | MAP1LC3A | microtubule associated protein 1 light chain 3 alpha | 1.16 |
| ENSG00000103154 | NECAB2 | N-terminal EF-hand calcium binding protein 2 | 1.13 |
| ENSG00000104903 | LYL1 | LYL1 basic helix-loop-helix family member | 1.04 |
| ENSG00000105701 | FKBP8 | FKBP prolyl isomerase 8 | 1.36 |
| ENSG00000105711 | SCN1B | sodium voltage-gated channel beta subunit 1 | 1.43 |
| ENSG00000105835 | NAMPT | nicotinamide phosphoribosyltransferase | 1.03 |
| ENSG00000109272 | PF4V1 | platelet factor 4 variant 1 | 1.50 |
| ENSG00000110203 | FOLR3 | folate receptor 3 | 1.94 |
| ENSG00000112053 | SLC26A8 | solute carrier family 26 member 8 | 1.58 |
| ENSG00000113140 | SPARC | secreted protein acidic and cysteine rich | 1.09 |
| ENSG00000113303 | BTNL8 | butyrophilin like 8 | 1.15 |
| ENSG00000114270 | COL7A1 | collagen type VII alpha 1 chain | 1.10 |
| ENSG00000115590 | IL1R2 | interleukin 1 receptor type 2 | 1.09 |
| ENSG00000115594 | IL1R1 | interleukin 1 receptor type 1 | 1.30 |
| ENSG00000116473 | RAP1A | RAP1A, member of RAS oncogene family [Source:HGNC Symbol;Acc:HGNC:9855] | 1.33 |
| ENSG00000120051 | CFAP58 | cilia and flagella associated protein 58 | 1.31 |
| ENSG00000120217 | CD274 | CD274 molecule | 1.17 |
| ENSG00000120885 | CLU | clusterin | 1.02 |
| ENSG00000122026 | RPL21 | ribosomal protein L21 | -1.36 |
| ENSG00000122432 | SPATA1 | spermatogenesis associated 1 | 1.04 |
| ENSG00000123610 | TNFAIP6 | TNF alpha induced protein 6 | 1.74 |
| ENSG00000124107 | SLPI | secretory leukocyte peptidase inhibitor | 1.01 |
| ENSG00000124256 | ZBP1 | Z-DNA binding protein 1 | 1.06 |
| ENSG00000125148 | MT2A | metallothionein 2A | 1.09 |
| ENSG00000126262 | FFAR2 | free fatty acid receptor 2 | 1.07 |
| ENSG00000126709 | IFI6 | interferon alpha inducible protein 6 | 1.65 |
| ENSG00000128383 | APOBEC3A | apolipoprotein B mRNA editing enzyme catalytic subunit 3A | 1.16 |
| ENSG00000128512 | DOCK4 | dedicator of cytokinesis 4 | 1.22 |
| ENSG00000128918 | ALDH1A2 | aldehyde dehydrogenase 1 family member A2 | 1.10 |
| ENSG00000129673 | AANAT | aralkylamine N-acetyltransferase | 1.17 |
| ENSG00000130489 | SCO2 | NA | 1.36 |
| ENSG00000132530 | XAF1 | XIAP associated factor 1 | 1.18 |
| ENSG00000134321 | RSAD2 | radical S-adenosyl methionine domain containing 2 | 2.29 |
| ENSG00000134548 | SPX | spexin hormone | 1.07 |
| ENSG00000134571 | MYBPC3 | myosin binding protein C, cardiac [Source:HGNC Symbol;Acc:HGNC:7551] | 1.13 |
| ENSG00000134668 | SPOCD1 | SPOC domain containing 1 | 1.14 |
| ENSG00000134686 | PHC2 | polyhomeotic homolog 2 | 1.12 |
| ENSG00000135114 | OASL | 2'-5'-oligoadenylate synthetase like | 1.67 |
| ENSG00000136689 | IL1RN | interleukin 1 receptor antagonist | 1.19 |
| ENSG00000136732 | GYPC | glycophorin C (Gerbich blood group) | 1.46 |
| ENSG00000137198 | GMPR | guanosine monophosphate reductase | 1.32 |
| ENSG00000137331 | IER3 | immediate early response 3 | 1.01 |
| ENSG00000137441 | FGFBP2 | fibroblast growth factor binding protein 2 | 1.07 |
| ENSG00000137507 | LRRC32 | leucine rich repeat containing 32 | 1.01 |
| ENSG00000138772 | ANXA3 | annexin A3 | 1.25 |
| ENSG00000139890 | REM2 | RRAD and GEM like GTPase 2 | 1.09 |
| ENSG00000140932 | CMTM2 | CKLF like MARVEL transmembrane domain containing 2 | 1.20 |
| ENSG00000141298 | SSH2 | slingshot protein phosphatase 2 | 2.36 |
| ENSG00000141404 | GNAL | G protein subunit alpha L | 1.05 |
| ENSG00000142552 | RCN3 | reticulocalbin 3 | 1.05 |
| ENSG00000143858 | SYT2 | synaptotagmin 2 | 1.28 |
| ENSG00000145736 | GTF2H2 | general transcription factor IIH subunit 2 | 1.12 |
| ENSG00000148926 | ADM | adrenomedullin | 1.53 |
| ENSG00000149131 | SERPING1 | serpin family G member 1 | 1.69 |
| ENSG00000152766 | ANKRD22 | ankyrin repeat domain 22 | 1.27 |
| ENSG00000153814 | JAZF1 | JAZF zinc finger 1 | 1.05 |
| ENSG00000154146 | NRGN | neurogranin | 1.02 |
| ENSG00000154188 | ANGPT1 | angiopoietin 1 | 1.21 |
| ENSG00000157542 | KCNJ6 | potassium voltage-gated channel subfamily J member 6 | 1.04 |
| ENSG00000157551 | KCNJ15 | potassium voltage-gated channel subfamily J member 15 | 1.24 |
| ENSG00000158050 | DUSP2 | dual specificity phosphatase 2 | 1.08 |
| ENSG00000158292 | GPR153 | G protein-coupled receptor 153 | 1.03 |
| ENSG00000159588 | CCDC17 | coiled-coil domain containing 17 | 1.28 |
| ENSG00000161040 | FBXL13 | F-box and leucine rich repeat protein 13 | 1.28 |
| ENSG00000161911 | TREML1 | triggering receptor expressed on myeloid cells like 1 | 1.16 |
| ENSG00000162366 | PDZK1IP1 | PDZK1 interacting protein 1 | 1.66 |
| ENSG00000162747 | FCGR3B | Fc fragment of IgG receptor IIIb | 1.17 |
| ENSG00000163464 | CXCR1 | C-X-C motif chemokine receptor 1 | 1.05 |
| ENSG00000163735 | CXCL5 | C-X-C motif chemokine ligand 5 | 1.48 |
| ENSG00000163736 | PPBP | pro-platelet basic protein | 1.40 |
| ENSG00000163737 | PF4 | platelet factor 4 | 1.72 |
| ENSG00000163739 | CXCL1 | C-X-C motif chemokine ligand 1 | 1.13 |
| ENSG00000163803 | PLB1 | phospholipase B1 | 1.20 |
| ENSG00000165181 | C9orf84 | shortage in chiasmata 1 | 1.41 |
| ENSG00000166091 | CMTM5 | CKLF like MARVEL transmembrane domain containing 5 | 1.23 |
| ENSG00000166546 | BEAN1 | brain expressed associated with NEDD4 1 | 1.03 |
| ENSG00000167434 | CA4 | carbonic anhydrase 4 | 1.49 |
| ENSG00000167536 | DHRS13 | dehydrogenase/reductase 13 | 1.03 |
| ENSG00000167676 | PLIN4 | perilipin 4 | 1.32 |
| ENSG00000167705 | RILP | Rab interacting lysosomal protein | 1.06 |
| ENSG00000167874 | TMEM88 | transmembrane protein 88 | 1.20 |
| ENSG00000167992 | VWCE | von Willebrand factor C and EGF domains | 1.96 |
| ENSG00000168062 | BATF2 | basic leucine zipper ATF-like transcription factor 2 | 1.65 |
| ENSG00000169247 | SH3TC2 | SH3 domain and tetratricopeptide repeats 2 | 1.08 |
| ENSG00000169704 | GP9 | glycoprotein IX platelet | 1.19 |
| ENSG00000169902 | TPST1 | tyrosylprotein sulfotransferase 1 | 1.46 |
| ENSG00000170956 | CEACAM3 | carcinoembryonic antigen related cell adhesion molecule 3 | 1.20 |
| ENSG00000171051 | FPR1 | formyl peptide receptor 1 | 1.03 |
| ENSG00000172243 | CLEC7A | C-type lectin domain containing 7A | 1.06 |
| ENSG00000175003 | SLC22A1 | solute carrier family 22 member 1 | 1.06 |
| ENSG00000175899 | A2M | alpha-2-macroglobulin | 1.07 |
| ENSG00000176788 | BASP1 | brain abundant membrane attached signal protein 1 | 1.04 |
| ENSG00000177989 | ODF3B | outer dense fiber of sperm tails 3B | 1.06 |
| ENSG00000178814 | OPLAH | 5-oxoprolinase, ATP-hydrolysing [Source:HGNC Symbol;Acc:HGNC:8149] | 1.05 |
| ENSG00000180089 | TMEM86B | transmembrane protein 86B | 1.06 |
| ENSG00000181016 | LSMEM1 | leucine rich single-pass membrane protein 1 | 1.27 |
| ENSG00000181778 | TMEM252 | transmembrane protein 252 | 1.05 |
| ENSG00000182310 | SPACA6 | sperm acrosome associated 6 | 1.66 |
| ENSG00000182732 | RGS6 | regulator of G protein signaling 6 | 1.23 |
| ENSG00000182885 | ADGRG3 | adhesion G protein-coupled receptor G3 | 1.07 |
| ENSG00000183762 | KREMEN1 | kringle containing transmembrane protein 1 | 1.54 |
| ENSG00000184500 | PROS1 | protein S | 1.05 |
| ENSG00000184557 | SOCS3 | suppressor of cytokine signaling 3 | 1.19 |
| ENSG00000184611 | KCNH7 | potassium voltage-gated channel subfamily H member 7 | 1.32 |
| ENSG00000185201 | IFITM2 | interferon induced transmembrane protein 2 | 1.34 |
| ENSG00000185442 | FAM174B | family with sequence similarity 174 member B | 1.30 |
| ENSG00000185507 | IRF7 | interferon regulatory factor 7 | 1.12 |
| ENSG00000187608 | ISG15 | ISG15 ubiquitin like modifier | 1.90 |
| ENSG00000187775 | DNAH17 | dynein axonemal heavy chain 17 | 1.27 |
| ENSG00000188056 | TREML4 | triggering receptor expressed on myeloid cells like 4 | 1.14 |
| ENSG00000188313 | PLSCR1 | phospholipid scramblase 1 | 1.05 |
| ENSG00000196358 | NTNG2 | netrin G2 | 1.26 |
| ENSG00000196684 | HSH2D | hematopoietic SH2 domain containing | 1.03 |
| ENSG00000198216 | CACNA1E | calcium voltage-gated channel subunit alpha1 E | 1.18 |
| ENSG00000198336 | MYL4 | myosin light chain 4 | 1.81 |
| ENSG00000198478 | SH3BGRL2 | SH3 domain binding glutamate rich protein like 2 | 1.02 |
| ENSG00000198736 | MSRB1 | methionine sulfoxide reductase B1 | 1.01 |
| ENSG00000198814 | GK | glycerol kinase | 1.27 |
| ENSG00000203804 | ADAMTSL4-AS1 | ADAMTSL4 antisense RNA 1 | 1.10 |
| ENSG00000203814 | HIST2H2BF | histone cluster 2 H2B family member f | 1.22 |
| ENSG00000203999 | LINC01270 | long intergenic non-protein coding RNA 1270 | 1.03 |
| ENSG00000204420 | C6orf25 | megakaryocyte and platelet inhibitory receptor G6b | 1.06 |
| ENSG00000205309 | NT5M | 5',3'-nucleotidase, mitochondrial [Source:HGNC Symbol;Acc:HGNC:15769] | 1.11 |
| ENSG00000205639 | MFSD2B | major facilitator superfamily domain containing 2B | 1.12 |
| ENSG00000205786 | LINC01531 | long intergenic non-protein coding RNA 1531 | 1.77 |
| ENSG00000205890 | RP11-473M20.5 | novel transcript, antisense to CCDC64B | 1.10 |
| ENSG00000206172 | HBA1 | hemoglobin subunit alpha 1 | 1.76 |
| ENSG00000206177 | HBM | hemoglobin subunit mu | 1.84 |
| ENSG00000211459 | MT-RNR1 | mitochondrially encoded 12S rRNA | 1.05 |
| ENSG00000211662 | IGLV3-21 | immunoglobulin lambda variable 3-21 | -1.62 |
| ENSG00000211947 | IGHV3-21 | immunoglobulin heavy variable 3-21 | -1.00 |
| ENSG00000211964 | IGHV3-48 | immunoglobulin heavy variable 3-48 | -1.21 |
| ENSG00000213347 | MXD3 | MAX dimerization protein 3 | 1.04 |
| ENSG00000213937 | CLDN9 | claudin 9 | 1.18 |
| ENSG00000214456 | PLIN5 | perilipin 5 | 1.38 |
| ENSG00000214872 | SMTNL1 | smoothelin like 1 | 2.16 |
| ENSG00000223722 | RP11-467L13.5 | interferon induced transmembrane protein 3 (1-8U) (IFITM3) pseudogene | 1.70 |
| ENSG00000223855 | HRAT92 | novel transcript | 1.32 |
| ENSG00000223935 | AC008074.3 | LGALSL divergent transcript | 1.01 |
| ENSG00000224650 | IGHV3-74 | immunoglobulin heavy variable 3-74 | -1.10 |
| ENSG00000225101 | OR52K3P | olfactory receptor family 52 subfamily K member 3 pseudogene | 1.72 |
| ENSG00000225217 | HSPA7 | heat shock protein family A (Hsp70) member 7 | 1.29 |
| ENSG00000225873 | LINC00694 | chromosome 3 open reading frame 86 | 1.40 |
| ENSG00000228315 | GUSBP11 | GUSB pseudogene 11 | 1.21 |
| ENSG00000231233 | CFAP58-AS1 | CFAP58 divergent transcript | 1.24 |
| ENSG00000232254 | CSF2RBP1 | colony stimulating factor 2 receptor beta common subunit pseudogene 1 | 1.05 |
| ENSG00000233429 | HOTAIRM1 | HOXA transcript antisense RNA, myeloid-specific 1 [Source:HGNC Symbol;Acc:HGNC:37117] | 1.49 |
| ENSG00000234998 | RP11-439A17.10 | novel transcript, antisense to FCGR1B | 1.15 |
| ENSG00000235169 | SMIM1 | small integral membrane protein 1 (Vel blood group) | 1.60 |
| ENSG00000235453 | TOPORS-AS1 | small integral membrane protein 27 | 1.13 |
| ENSG00000238243 | OR2W3 | olfactory receptor family 2 subfamily W member 3 | 1.67 |
| ENSG00000243797 | CTB-111H14.1 | novel transcript, sense overlapping CCDC71L | 1.69 |
| ENSG00000244617 | ASPRV1 | aspartic peptidase retroviral like 1 | 1.34 |
| ENSG00000244682 | FCGR2C | Fc fragment of IgG receptor IIc (gene/pseudogene) | 1.05 |
| ENSG00000248099 | INSL3 | insulin like 3 | 1.08 |
| ENSG00000248485 | PCP4L1 | Purkinje cell protein 4 like 1 | 1.01 |
| ENSG00000249456 | RP11-298J20.4 | novel transcript, sense overlapping ZRANB1 | 1.16 |
| ENSG00000249476 | CTD-2587M2.1 | novel transcript | 1.01 |
| ENSG00000250334 | LINC00989 | long intergenic non-protein coding RNA 989 | 1.09 |
| ENSG00000250616 | RP11-455F5.3 | novel transcript, antisense to YPEL3 | 1.02 |
| ENSG00000255557 | RP11-770G2.2 | novel transcript | 1.41 |
| ENSG00000256249 | RP11-324E6.6 | novel transcript | 1.04 |
| ENSG00000258344 | RP11-968A15.8 | novel transcript, sense overlapping to COPZ1 & HNRNPA1 | 1.03 |
| ENSG00000258484 | SPESP1 | sperm equatorial segment protein 1 | 1.26 |
| ENSG00000258867 | LINC01146 | long intergenic non-protein coding RNA 1146 | 1.04 |
| ENSG00000259448 | RP11-16E12.1 | long intergenic non-protein coding RNA 2352 | 1.01 |
| ENSG00000260528 | FAM157C | family with sequence similarity 157 member C | 2.86 |
| ENSG00000260911 | RP11-196G11.2 | novel transcript | 1.14 |
| ENSG00000268861 | CTD-2207O23.3 | Rho/Rac guanine nucleotide exchange factor 18 | 1.17 |
| ENSG00000272888 | LINC01578 | long intergenic non-protein coding RNA 1578 | 1.15 |
| ENSG00000273112 | RP11-25K21.6 | novel transcript, readthrough between FCGR2A and FCGR2C polymormphic pseudogene | 1.09 |
| ENSG00000274290 | HIST1H2BE | histone cluster 1 H2B family member e | 1.11 |
| ENSG00000275395 | FCGBP | Fc fragment of IgG binding protein | -1.06 |
| ENSG00000276241 | CTB-91J4.1 | novel transcript, antisense TBC1D3B | 1.33 |
| ENSG00000277855 | RP11-154H23.4 | novel transcript, antisense to FOXP1 | 1.04 |
| ENSG00000282804 | RP3-369A17.6 | novel protein | 1.18 |

Supplementary Table 3. Genes in the exposure signature in this study that have been reported in other studies with active TB, latent and incipient TB. Numbers in cells are log2 fold change relative to comparison group (negative value represents downregulation); +, gene present but fold change not reported; -, gene not reported in study; *, IGRA positive vs IGRA-negative non-exposed controls; **, IGRA/TST positive progressors vs non-progressors.

| Our exposure genes | | Active TB signatures (Active TB vs healthy controls/IGRA negative) | | | | | | | Active TB signatures (Active TB vs Latent/ IGRA positive) | | | | | | | | | Latent * | Incipient ** |
| --- | --- | --- | --- | --- | --- | --- | --- | --- | --- | --- | --- | --- | --- | --- | --- | --- | --- | --- | --- |
| Gene | Contacts (this study) | Active TB (this study) | Berry ^1^ | Bloom ^2^ | Singhania London cohort ^3^ | Ottenhoff ^4^ | Lee ^5^ | Sambarey ^6^ | Anderson ^7^ | Kaforou ^8^ | Lee ^5^ | Bah ^9^ | Leong ^10^ | Singhania London dataset ^3^ | Singhania/ Berry Combined dataset ^3^ | Singhania Leicester dataset ^3^ | Gideon ^11^ | Lee ^5^ | Zak ^12^ |
| ADM | 1.53 | 2.55 | + | + | 1.3 | - | 2.0 | - | - | - | 4.0 | - | - | 0.9 | - | - | - | -2.0 | - |
| ANKRD22 | 1.27 | 4.14 | + | + | 4.2 | - | - | - | - | UP | - | - | 2.4 | 3.5 | 4.6 | 4.7 | - | - | + |
| ANXA3 | 1.25 | 2.90 | + | + | 1.2 | - | - | - | - | - | - | - | - | 1.1 | - | - | - | - | - |
| ASPRV1 | 1.34 | 1.60 | - | - | 1.2 | - | - | - | - | - | - | 1.6 | - | 0.7 | - | - | - | - | - |
| BATF2 | 1.65 | 3.58 | + | + | 3.3 | + | - | - | - | - | - | - | 2.6 | 2.2 | 3.8 | 3.9 | - | - | + |
| BLVRB | 1.16 | - | - | - | - | - | - | - | - | - | - | - | - | - | - | - | - | - | - |
| C6orf25 | 1.06 | 1.28 | - | + | - | - | - | - | - | - | - | - | - | - | - | - | - | - | - |
| CA4 | 1.49 | 2.60 | - | + | - | - | - | - | - | - | - | - | - | - | - | - | - | - | - |
| CACNA1E | 1.18 | 2.80 | + | + | 2.1 | - | - | - | - | - | - | - | - | 1.5 | 2.2 | 2.4 | - | - | - |
| CD274 | 1.17 | 3.30 | + | + | 2.9 | - | - | - | - | - | - | - | 1.9 | 2.1 | 3.0 | 3.1 | - | - | - |
| CEACAM3 | 1.20 | 1.78 | - | - | - | - | - | - | - | - | - | 1.6 | - | - | - | - | - | - | - |
| CLDN9 | 1.18 | 1.73 | - | - | - | - | - | 1.3 | - | - | - | - | - | - | - | - | - | - | - |
| CMTM5 | 1.23 | 1.57 | - | + | - | - | - | - | - | - | - | 1.6 | - | - | - | - | - | - | - |
| CXCL1 | 1.13 | 1.43 | - | - | - | - | - | - | - | - | - | - | - | - | - | - | - | - | - |
| DOCK4 | 1.22 | 1.96 | - | - | 1.4 | - | - | - | - | - | - | - | - | 0.8 | - | - | - | - | - |
| DUSP2 | 1.08 | - | - | - | -0.3 | - | - | - | - | - | - | - | - | -1.1 | - | - | - | -1.9 | - |
| FCGBP | -1.06 | -2.28 | + | + | -1.2 | - | - | - | - | - | - | - | - | -1.3 | - | - | - | - | - |
| FCGR2C | 1.05 | 1.22 | - | + | - | - | - | - | - | - | - | - | - | - | - | - | - | - | - |
| FCGR3B | 1.17 | 1.97 | - | + | 1.0 | - | - | - | - | - | - | - | - | 0.7 | - | - | - | - | - |
| FFAR2 | 1.07 | 1.65 | + | + | - | - | - | - | - | - | 1.6 | 1.6 | - | - | - | - | - | - | - |
| FGFBP2 | 1.07 | - | - | - | - | - | -1.1 | - | - | - | - | -1.6 | - | - | - | - | - | - | - |
| FOLR3 | 1.94 | 3.05 | - | - | - | - | - | 2.5 | - | - | - | - | - | - | - | - | - | - | - |
| FPR1 | 1.03 | 1.58 | - | - | - | - | - | - | - | - | - | - | - | - | - | - | 1.5 | - | - |
| GK | 1.27 | 2.10 | + | + | 1.3 | - | - | - | - | - | - | - | - | 1.0 | - | - | - | - | - |
| GP9 | 1.19 | 1.24 | - | + | - | - | - | - | - | - | - | - | - | - | - | - | - | - | - |
| HIST1H2BE | 1.11 | 2.59 | - | + | - | - | - | - | - | - | - | - | - | - | - | - | - | - | - |
| HIST2H2BF | 1.22 | 2.68 | + | - | 1.2 | - | - | - | - | - | - | - | - | 0.9 | - | - | - | - | - |
| IER3 | 1.01 | 1.13 | - | + | - | - | - | - | - | - | - | - | - | - | - | - | - | -1.7 | - |
| IFI6 | 1.65 | 1.66 | + | + | 1.3 | - | - | - | - | - | - | - | - | 0.6 | 2.0 | 2.0 | 1.9 | - | - |
| IFITM2 | 1.34 | 1.78 | - | - | - | - | - | - | - | - | - | - | - | - | - | - | 1.6 | - | - |
| IL1RN | 1.19 | 1.54 | + | + | - | - | - | - | - | - | 1.1 | - | - | - | - | - | - | - | - |
| INSL3 | 1.08 | 1.52 | - | - | - | - | - | 1.1 | - | - | - | - | - | - | - | - | - | - | - |
| IRF7 | 1.12 | 1.36 | + | + | 1.1 | - | - | - | - | - | - | 1.6 | - | 0.7 | 1.2 | 1.8 | 2.0 | - | - |
| ISG15 | 1.90 | 1.83 | + | - | 1.9 | - | - | - | - | - | - | 1.7 | - | 0.5 | 2.0 | 2.2 | - | - | - |
| ITGA2B | 1.23 | 2.13 | - | + | 1.2 | - | - | - | - | - | - | - | - | 1.3 | - | - | 1.5 | - | - |
| KCNJ15 | 1.24 | 2.37 | + | + | 1.3 | - | - | - | - | - | - | - | - | 1.4 | - | - | - | - | - |
| KREMEN1 | 1.54 | 3.65 | + | + | 2.0 | - | - | - | - | - | - | - | - | 1.7 | - | - | - | - | - |
| LINC01146 | 1.04 | - | - | - | - | - | - | 1.5 | - | - | - | - | - | - | - | - | - | - | - |
| LINC01270 | 1.03 | 1.87 | - | - | - | - | - | 1.3 | - | - | - | - | - | - | - | - | - | - | - |
| MT2A | 1.09 | 1.15 | - | - | 1.3 | - | - | - | - | - | - | - | 1.3 | 0.5 | 1.2 | 1.9 | - | - | - |
| MYBPC3 | 1.13 | 1.93 | - | - | - | - | - | 1.4 | - | - | - | - | - | - | - | - | - | - | - |
| NAMPT | 1.03 | 1.82 | - | + | - | - | - | - | - | - | - | - | - | - | - | - | - | - | - |
| NTNG2 | 1.26 | 2.12 | - | - | 1.1 | - | - | - | - | - | 1.1 | - | - | 0.6 | - | - | - | - | - |
| OASL | 1.67 | 1.64 | + | - | 1.3 | - | - | - | - | - | - | - | - | 0.4 | 1.5 | 1.8 | 2.0 | - | - |
| ODF3B | 1.06 | 1.97 | - | + | 1.7 | - | - | - | - | - | - | - | - | 1.0 | - | - | - | - | - |
| OR52K3P | 1.72 | 2.45 | - | - | - | - | - | - | - | - | - | 1.6 | - | - | - | - | - | - | - |
| OSM | 1.23 | 2.01 | + | + | 1.2 | - | - | - | - | - | - | 1.8 | - | 0.8 | - | - | - | -3.5 | - |
| PF4V1 | 1.50 | 1.76 | - | + | - | - | - | - | - | - | - | - | - | - | - | - | - | - | - |
| PGLYRP1 | 1.21 | 2.87 | + | - | 1.1 | - | - | - | - | - | - | - | - | 1.0 | - | - | - | - | - |
| PLIN4 | 1.32 | 1.98 | - | - | - | - | - | 1.2 | - | - | - | - | - | - | - | - | - | - | - |
| PLSCR1 | 1.05 | 1.69 | + | + | 1.4 | - | - | - | - | - | - | - | - | 1.0 | 1.8 | 1.7 | - | - | - |
| PROS1 | 1.05 | 2.38 | - | + | 0.8 | - | - | - | - | - | - | - | - | 1.0 | - | - | - | - | - |
| RAP1A | 1.33 | - | - | - | - | - | - | - | + | - | - | - | - | - | - | - | - | - | - |
| RILP | 1.06 | - | - | - | - | - | - | - | - | - | 1.0 | - | - | - | - | - | - | - | - |
| RSAD2 | 2.29 | 2.39 | + | - | 2.1 | - | - | - | - | - | - | - | - | 0.6 | 3.1 | 2.5 | - | - | - |
| SCARF1 | 1.06 | 2.51 | - | + | 1.7 | - | - | - | - | - | - | 1.7 | - | 1.5 | - | - | - | - | + |
| SCN1B | 1.43 | 1.89 | - | - | - | - | - | 1.1 | - | - | - | - | - | - | - | - | - | - | - |
| SCO2 | 1.36 | 2.44 | + | + | 1.8 | - | - | - | - | - | - | 1.9 | - | 1.2 | 1.8 | 2.4 | - | - | - |
| SERPING1 | 1.69 | 3.49 | + | + | 3.1 | - | 1.9 | - | - | - | - | - | 2.2 | 2.0 | 3.8 | 3.8 | - | - | + |
| SLC26A8 | 1.58 | 3.07 | + | + | 1.5 | - | - | 1.1 | - | - | - | - | - | 1.4 | 2.2 | 2.1 | - | - | - |
| SOCS3 | 1.19 | 2.24 | + | + | 1.3 | - | - | 1.2 | - | - | - | - | - | 0.8 | - | - | - | -1.1 | - |
| SPARC | 1.09 | 1.38 | - | + | - | - | - | - | - | - | - | - | - | - | - | - | - | - | - |
| TMEM88 | 1.20 | 1.85 | - | - | - | - | - | 1.2 | - | - | - | - | - | - | - | - | - | - | - |
| TNFAIP6 | 1.74 | 2.74 | + | + | 1.9 | - | - | - | - | - | - | - | - | 1.2 | - | - | 2.0 | - | - |
| TOPORS-AS1 | 1.13 | 1.17 | - | - | - | - | - | - | - | - | - | - | 0.7 | - | - | - | - | - | - |
| TPST1 | 1.46 | 1.56 | - | - | - | - | - | - | - | - | - | 1.5 | - | - | - | - | - | - | - |
| TREML1 | 1.16 | 1.66 | - | + | - | - | - | - | - | - | - | 1.7 | - | - | - | - | - | - | - |
| XAF1 | 1.18 | 1.50 | + | + | 1.3 | - | -1.2 | - | - | - | - | 1.7 | - | 0.8 | 1.8 | 1.8 | - | - | - |
| ZBP1 | 1.06 | 1.52 | + | + | 1.2 | - | - | - | - | - | - | - | - | 0.5 | 1.2 | 1.4 | - | - | - |

Supplementary Table 4. All overrepresented GO Biological Processes of the protein-protein network from STRING analysis. The three highlighted pathways are represented as colored circles in Figure 1.

| GO term ID | GO (Biological Processes) description | false discovery rate |
| --- | --- | --- |
| GO:0006955 | immune response | 9.51E-11 |
| GO:0002376 | immune system process | 3.25E-10 |
| GO:0006952 | defense response | 6.26E-09 |
| GO:0001775 | cell activation | 1.88E-06 |
| GO:0045055 | regulated exocytosis | 6.85E-06 |
| GO:0002252 | immune effector process | 1.29E-05 |
| GO:0098542 | defense response to other organism | 1.66E-05 |
| GO:0051707 | response to other organism | 2.56E-05 |
| GO:0019221 | cytokine-mediated signaling pathway | 2.91E-05 |
| GO:0032940 | secretion by cell | 4.48E-05 |
| GO:0034097 | response to cytokine | 4.48E-05 |
| GO:0045071 | negative regulation of viral genome replication | 4.48E-05 |
| GO:0002443 | leukocyte mediated immunity | 5.34E-05 |
| GO:1902624 | positive regulation of neutrophil migration | 6.59E-05 |
| GO:0009605 | response to external stimulus | 0.00011 |
| GO:0045087 | innate immune response | 0.00012 |
| GO:0060337 | type I interferon signaling pathway | 0.00012 |
| GO:0019730 | antimicrobial humoral response | 0.00019 |
| GO:0002684 | positive regulation of immune system process | 0.00029 |
| GO:0045321 | leukocyte activation | 0.00034 |
| GO:0002682 | regulation of immune system process | 0.00041 |
| GO:0090023 | positive regulation of neutrophil chemotaxis | 0.00044 |
| GO:0002446 | neutrophil mediated immunity | 0.00049 |
| GO:0002274 | myeloid leukocyte activation | 0.00057 |
| GO:0002576 | platelet degranulation | 0.00058 |
| GO:0006950 | response to stress | 0.00059 |
| GO:0030335 | positive regulation of cell migration | 0.00062 |
| GO:0032101 | regulation of response to external stimulus | 0.00067 |
| GO:0060326 | cell chemotaxis | 0.00077 |
| GO:0006954 | inflammatory response | 0.0011 |
| GO:0043312 | neutrophil degranulation | 0.0011 |
| GO:0016192 | vesicle-mediated transport | 0.0012 |
| GO:0006959 | humoral immune response | 0.0013 |
| GO:0050896 | response to stimulus | 0.0014 |
| GO:0070098 | chemokine-mediated signaling pathway | 0.0014 |
| GO:0048583 | regulation of response to stimulus | 0.0015 |
| GO:0050776 | regulation of immune response | 0.0015 |
| GO:0042832 | defense response to protozoan | 0.0016 |
| GO:0009615 | response to virus | 0.0017 |
| GO:0030168 | platelet activation | 0.0017 |
| GO:2000659 | regulation of interleukin-1-mediated signaling pathway | 0.0017 |
| GO:0031347 | regulation of defense response | 0.002 |
| GO:0050727 | regulation of inflammatory response | 0.0021 |
| GO:0002687 | positive regulation of leukocyte migration | 0.0022 |
| GO:0002366 | leukocyte activation involved in immune response | 0.0024 |
| GO:0007596 | blood coagulation | 0.0026 |
| GO:0002673 | regulation of acute inflammatory response | 0.0029 |
| GO:0006935 | chemotaxis | 0.003 |
| GO:0010646 | regulation of cell communication | 0.0045 |
| GO:0050778 | positive regulation of immune response | 0.0048 |
| GO:0009966 | regulation of signal transduction | 0.0053 |
| GO:0023051 | regulation of signaling | 0.0054 |
| GO:0061844 | antimicrobial humoral immune response mediated by antimicrobial peptide | 0.0056 |
| GO:0001817 | regulation of cytokine production | 0.007 |
| GO:0009617 | response to bacterium | 0.0089 |
| GO:0001869 | negative regulation of complement activation, lectin pathway | 0.009 |
| GO:0034157 | positive regulation of toll-like receptor 7 signaling pathway | 0.009 |
| GO:0007597 | blood coagulation, intrinsic pathway | 0.0108 |
| GO:0032879 | regulation of localization | 0.0112 |
| GO:0002697 | regulation of immune effector process | 0.0115 |
| GO:0010469 | regulation of signaling receptor activity | 0.0115 |
| GO:0045088 | regulation of innate immune response | 0.0115 |
| GO:0050900 | leukocyte migration | 0.0115 |
| GO:0051704 | multi-organism process | 0.0115 |
| GO:0051607 | defense response to virus | 0.0123 |
| GO:0030334 | regulation of cell migration | 0.0139 |
| GO:0006810 | transport | 0.0156 |
| GO:0042742 | defense response to bacterium | 0.016 |
| GO:0048585 | negative regulation of response to stimulus | 0.0162 |
| GO:0098657 | import into cell | 0.0169 |
| GO:0030449 | regulation of complement activation | 0.0176 |
| GO:0042060 | wound healing | 0.0176 |
| GO:0007166 | cell surface receptor signaling pathway | 0.0182 |
| GO:0051179 | localization | 0.0184 |
| GO:2000257 | regulation of protein activation cascade | 0.0193 |
| GO:0035456 | response to interferon-beta | 0.0195 |
| GO:0009611 | response to wounding | 0.02 |
| GO:0032649 | regulation of interferon-gamma production | 0.0204 |
| GO:0051048 | negative regulation of secretion | 0.0204 |
| GO:0051270 | regulation of cellular component movement | 0.022 |
| GO:0001959 | regulation of cytokine-mediated signaling pathway | 0.0226 |
| GO:2000660 | negative regulation of interleukin-1-mediated signaling pathway | 0.0226 |
| GO:0050878 | regulation of body fluid levels | 0.0229 |
| GO:0065008 | regulation of biological quality | 0.0243 |
| GO:0060368 | regulation of Fc receptor mediated stimulatory signaling pathway | 0.0284 |
| GO:0032103 | positive regulation of response to external stimulus | 0.0333 |
| GO:0045906 | negative regulation of vasoconstriction | 0.0352 |
| GO:0051902 | negative regulation of mitochondrial depolarization | 0.0352 |
| GO:1903523 | negative regulation of blood circulation | 0.0368 |
| GO:0032496 | response to lipopolysaccharide | 0.037 |
| GO:0070613 | regulation of protein processing | 0.0374 |
| GO:0002718 | regulation of cytokine production involved in immune response | 0.0378 |
| GO:0043900 | regulation of multi-organism process | 0.0385 |
| GO:0008015 | blood circulation | 0.0388 |
| GO:0048519 | negative regulation of biological process | 0.0388 |
| GO:1903555 | regulation of tumor necrosis factor superfamily cytokine production | 0.0396 |
| GO:0040011 | locomotion | 0.0403 |
| GO:0016064 | immunoglobulin mediated immune response | 0.0408 |
| GO:0051239 | regulation of multicellular organismal process | 0.0408 |
| GO:0042221 | response to chemical | 0.0415 |
| GO:1903531 | negative regulation of secretion by cell | 0.0415 |
| GO:0031348 | negative regulation of defense response | 0.0427 |
| GO:0048584 | positive regulation of response to stimulus | 0.0428 |
| GO:0072376 | protein activation cascade | 0.0428 |
| GO:0001818 | negative regulation of cytokine production | 0.0433 |
| GO:0051240 | positive regulation of multicellular organismal process | 0.044 |
| GO:0010033 | response to organic substance | 0.0441 |
| GO:0001819 | positive regulation of cytokine production | 0.0451 |

**REFERENCES**

1. Berry MP, Graham CM, McNab FW, Xu Z, Bloch SA, Oni T, et al. An interferon-inducible neutrophil-driven blood transcriptional signature in human tuberculosis. Nature. 2010;466(7309):973-7.

2. Bloom CI, Graham CM, Berry MP, Wilkinson KA, Oni T, Rozakeas F, et al. Detectable changes in the blood transcriptome are present after two weeks of antituberculosis therapy. PloS one. 2012;7(10):e46191.

3. Singhania A, Verma R, Graham CM, Lee J, Tran T, Richardson M, et al. A modular transcriptional signature identifies phenotypic heterogeneity of human tuberculosis infection. Nature Communications. 2018;9(1):2308.

4. Ottenhoff TH, Dass RH, Yang N, Zhang MM, Wong HE, Sahiratmadja E, et al. Genome-wide expression profiling identifies type 1 interferon response pathways in active tuberculosis. PLoS One. 2012;7(9):e45839.

5. Lee S-W, Wu LS-H, Huang G-M, Huang K-Y, Lee T-Y, Weng JT-Y, editors. Gene expression profiling identifies candidate biomarkers for active and latent tuberculosis. BMC bioinformatics; 2016: BioMed Central.

6. Sambarey A, Devaprasad A, Mohan A, Ahmed A, Nayak S, Swaminathan S, et al. Unbiased identification of blood-based biomarkers for pulmonary tuberculosis by modeling and mining molecular interaction networks. EBioMedicine. 2017;15:112-26.

7. Anderson ST, Kaforou M, Brent AJ, Wright VJ, Banwell CM, Chagaluka G, et al. Diagnosis of childhood tuberculosis and host RNA expression in Africa. New England Journal of Medicine. 2014;370(18):1712-23.

8. Kaforou M, Wright VJ, Oni T, French N, Anderson ST, Bangani N, et al. Detection of tuberculosis in HIV-infected and-uninfected African adults using whole blood RNA expression signatures: a case-control study. PLoS Med. 2013;10(10):e1001538.

9. Bah SY, Forster T, Dickinson P, Kampmann B, Ghazal P. Meta-analysis identification of highly robust and differential immune-metabolic signatures of systemic host response to acute and latent tuberculosis in children and adults. Frontiers in genetics. 2018;9:457.

10. Leong S, Zhao Y, Joseph NM, Hochberg NS, Sarkar S, Pleskunas J, et al. Existing blood transcriptional classifiers accurately discriminate active tuberculosis from latent infection in individuals from south India. Tuberculosis. 2018;109:41-51.

11. Gideon HP, Skinner JA, Baldwin N, Flynn JL, Lin PL. Early Whole Blood Transcriptional Signatures Are Associated with Severity of Lung Inflammation in Cynomolgus Macaques with Mycobacterium tuberculosis Infection. The Journal of Immunology. 2016;197(12):4817-28.

12. Zak DE, Penn-Nicholson A, Scriba TJ, Thompson E, Suliman S, Amon LM, et al. A blood RNA signature for tuberculosis disease risk: a prospective cohort study. The Lancet. 2016.
